# Parkinsonism in spinocerebellar ataxia with axonal neuropathy caused by adult-onset *COA7* variants: a case report

**DOI:** 10.1186/s12883-023-03202-w

**Published:** 2023-06-01

**Authors:** Shogo Ouchi, Kazuhiro Ishii, Kenjiro Kosaki, Hisato Suzuki, Mamiko Yamada, Toshiki Takenouchi, Akira Tamaoka

**Affiliations:** 1 Department of Neurology, Division of Clinical Medicine, Faculty of Medicine, University of Tsukuba, Ten’nudai 1-1-1, Tsukuba, Ibaraki 305-8575 Japan; 2 Center for Medical Genetics, Keio University, 35 Shinanomachi Shinju-Ku, Tokyo, 160-8582 Japan

**Keywords:** COA7, Parkinsonism, Spinocerebellar ataxia, Charcot-Marie-Tooth disease

## Abstract

**Background:**

Individuals with variants of cytochrome c oxidase assembly factor 7 (*COA7*), a mitochondrial functional-related gene, exhibit symptoms of spinocerebellar ataxia with axonal neuropathy before the age of 20. However, *COA7* variants with parkinsonism or adult-onset type cases have not been described.

**Case presentation:**

We report the case of a patient who developed cerebellar symptoms and slowly progressive sensory and motor neuropathy in the extremities, similar to Charcot-Marie-Tooth disease, at age 30, followed by parkinsonism at age 58. Exome analysis revealed *COA7* missense mutation in homozygotes (NM_023077.2:c.17A > G, NP_075565.2: p.Asp6Gly). Dopamine transporter single-photon emission computed tomography using a ^123^I-Ioflupane revealed clear hypo-accumulation in the bilateral striatum. However, ^123^I-metaiodobenzylguanidine myocardial scintigraphy showed normal sympathetic nerve function. Levodopa administration improved parkinsonism in this patient.

**Conclusions:**

*COA7* gene variants may have caused parkinsonism in this case because mitochondrial function-related genes, such as *parkin* and *PINK1,* are known causative genes in some familial Parkinson’s diseases.

## Background

The cytochrome c oxidase assembly factor 7 (*COA7*) encodes COA7 (NM_023077.2), a protein known as respiratory chain assembly factor 1, sel1 repeat-containing protein 1, and C1orf163. *COA7* variants cause mitochondrial dysfunction [1, 2].

Only six cases of *COA7* gene abnormalities have been reported. The neurological manifestations of spinocerebellar ataxia (SCA) with axonal neuropathy are based on cerebellar symptoms and axonal peripheral neuropathy and are accompanied by developmental disorders, cognitive impairment, and spastic paraplegia. Additionally, in all previously reported cases, symptoms occurred at young ages (≤ 15 years), and no cases of adult-onset (> 20 years) have been reported [1–3].

We report a case in which symptoms developed at the age of 30, with the patient exhibiting cerebellar symptoms with Charcot-Marie-Tooth disease (CMT)-like slowly progressive sensory and motor neuropathy in the extremities and parkinsonism. We also discuss the possible pathogenesis of *COA7* variants and dopa-responsive parkinsonism.

## Case presentation

A 60-year-old man presented with bradykinesia and gait disturbance. Atrophy predominantly developed in the distal muscles of his lower extremities at approximately 30 years of age. This atrophy slowly progressed, with mild instability when walking and a cramping sensation in both lower extremities at 53 years. At 54 years, a close examination revealed spastic paralysis in both lower limbs, CMT-like muscle weakness, muscle atrophy with distal muscle dominance owing to peripheral neuropathy, and cerebellar ataxia of the limbs. Brain magnetic resonance imaging (MRI) revealed mild cerebellar atrophy, and a nerve conduction study showed axonal-predominant peripheral neuropathy in both lower extremities. Therefore, a preliminary diagnosis of SCA with CMT was made. The patient noticed bradykinesia at age 58. There was no consanguinity within his family.

A neurological examination revealed no cognitive impairment (Mini-Mental State Examination score of 30 points), diplopia, and saccadic eye movements with smooth eye pursuit without nystagmus or oculomotor apraxia. Hypomimia, like a masked face, was observed without facial muscle weakness. Mild slurred speech and dysphagia were observed. Bilateral upper extremities had dysmetria and dysdiadochokinesia. Mild muscle weakness was observed in the bilateral distal lower extremity muscles, whereas other muscles were normal. Pes cavus was observed bilaterally, and tendon reflexes were normal, except for decreased bilateral Achilles tendon reflexes. No pathological reflexes were observed, including the Babinski reflexes. Bradykinesia and dystonia were observed in both hands. Muscle tonus showed muscle stiffness predominantly in the left lower extremity and mild clasp-knee-like phenomena in both knee joints. No other involuntary movements, including resting, postural, or action tremors, were observed. The sensory disturbance demonstrated a distal dominant pattern characterized by a glove-and-stocking-like distribution with decreasing proprioceptive, positional, and vibratory sensory perception. The Romberg test was positive, and the patient had a broad-based gait.

Routine blood and cerebrospinal fluid (CSF) examination results were normal. The liver and renal function were normal, and the creatine kinase level (84 U/L) was within the normal range. Serum autoantibody, syphilis antibody, human T-cell leukemia virus type 1 antibody, and vitamin B12 levels were normal. Glucose tolerance and thyroid and adrenal functions were normal. Blood lactate (8.4 mg/dL; normal range, 3.7–16.3) and pyruvate (0.63 mg/dL; normal range, 0.3–0.9) levels were normal; however, CSF lactate (21.9 mg/dL) and pyruvate (1.22 mg/dL) levels were increased.

Nerve conduction study results were normal in both upper extremities; however, the peroneal and sural nerves were not derivable, and the tibial nerve exhibited a mixed axonal demyelinating pattern (amplitude, 0.26 mV; velocity, 33.9 m/s) with temporal dispersion. Electromyography showed long-duration and high-amplitude muscle action potentials, indicating reinnervation as a chronic neurogenic change. Acute denervation findings, such as fasciculation and positive sharp waves, were not observed.

**Fig. 1:**
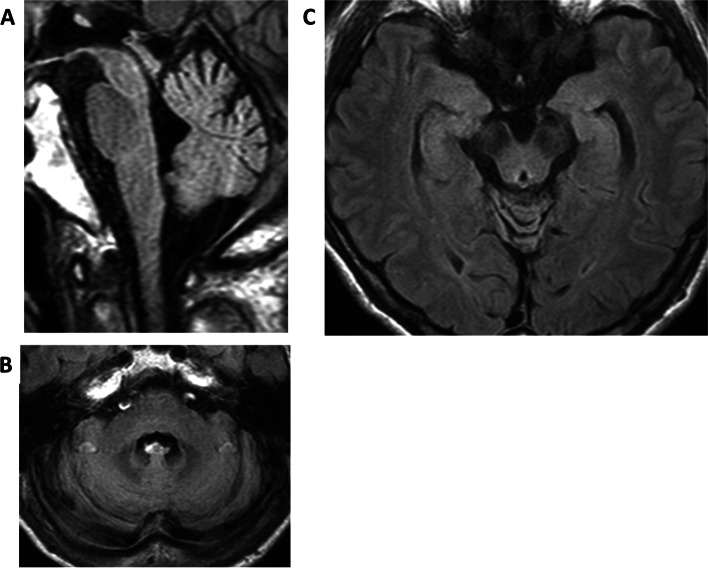
Brain magnetic resonance fluid-attenuated inversion recovery (MRI-FLAIR) images. The axial and sagittal views reveal mild atrophy of the cerebellar vermis and hemisphere, but no atrophy of the brain stem. **A**, **B** Atrophy of the cerebral cortex or hippocampus is not observed. (**C**)

**Fig. 2:**
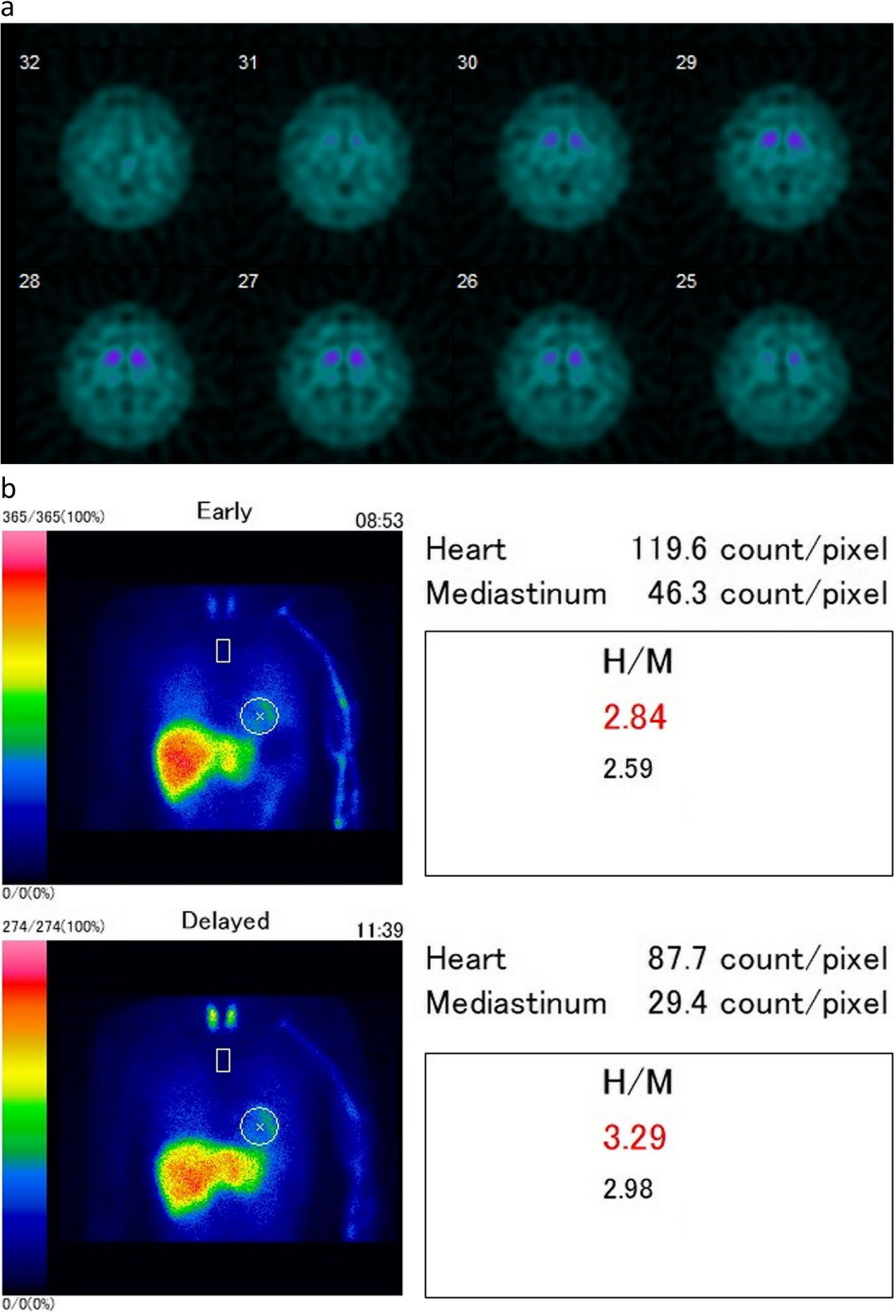
**a** Dopamine transporter (DAT) scan (dopamine transporter single-photon emission computed tomography [SPECT] using ^123^I-Ioflupane) shows a clear hypo-accumulation in the bilateral striatum with specific binding ratios (SBR) of 2.46 and 2.85 on the right and left sides, respectively. The asymmetric index was 14.8%. **b** ^123^I-metaiodobenzylguanidine (MIBG) myocardial scintigraphy showed that the heart-to-mediastinum (H/M) ratio was 2.84 (normal > 2.2) and 3.29 (normal > 2.2) in the early and late phases, respectively. The washout ratio was 15.3% (normal < 30%), and there was no evidence of myocardial sympathetic nerve dysfunction

Brain MRI revealed mild cerebellar atrophy in the vermis and hemispheric regions; however, no atrophy was observed in the cerebral cortex and brainstem (Fig. 1). No abnormalities, including cavity formation, were observed in the cerebral cortex or white matter. The dopamine transporter (DAT) scan (dopamine transporter single-photon emission computed tomography using ^123^I-Ioflupane) revealed decreased uptake in the bilateral striata (Fig. 2a). However, ^123^I-metaiodobenzylguanidine (MIBG) myocardial scintigraphy showed normal cardiac accumulation (Fig. 2b).

**Fig. 3:**
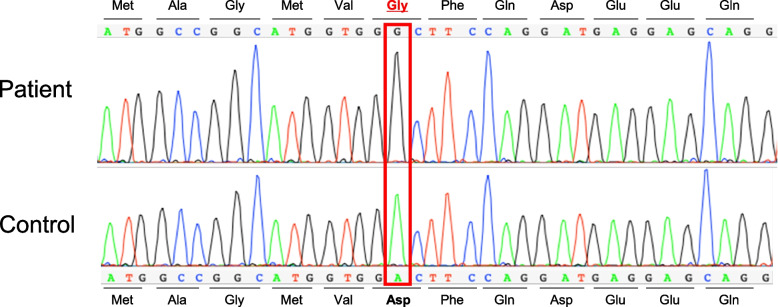
Deoxyribonucleic acid sequencing results using Sanger sequencing. The homozygous *COA7* gene missense mutation, c.17 A > G, is identified in this patient. The red frame indicates c.17 nucleotide. The wild-type *COA7* gene sequence is shown at the bottom of the figure as a control

An exome analysis was performed based on the Initiative on Rare and Undiagnosed Diseases project [4]. Genomic deoxyribonucleic acid was extracted from the peripheral blood leukocytes of the patient. Whole-exome sequencing in the patient was performed as described previously [5]. Briefly, all the exons were captured using the SureSelect All Exon V6 kit (Agilent Technologies, Santa Clara, CA). Subsequently, exome analyses were performed using the NovaSeq 6000 platform (Illumina, San Diego, CA). Sanger sequencing confirmed a previously reported missense mutation in the homozygous (NM_023077.2: c.17A > G, NP_075565.2: p.Asp6Gly) *COA7* gene (NM_023077.2) [1] (Fig. 3). No abnormal expansion related to SCAs, such as SCA1, SCA2, SCA3, SCA6, SCA8, SCA17, SCA31, and DRPLA, was observed in translational or non-translational regions that could be verified by a commercial testing company (BML, inc. Tokyo, JAPAN). We diagnosed the patient with SCA and axonal neuropathy type 3 caused by a missense *COA7* gene variant. Levodopa/carbidopa 300 mg (three times daily) was prescribed for parkinsonism, which caused a decrease in the patient’s daily activities (Modified Hoehn and Yahr Scale stage 2). The pre-treatment Movement Disorder Society-sponsored revision of the Unified Parkinson's Disease Rating Scale parts II and III score of 30 (part II, 8; part III, 22) improved to 22 (part II, 7; part III, 15) after levodopa administration [6]. This treatment was effective and continued.

## Discussion and Conclusions

Variations in some genes that encode proteins involved in mitochondrial function maintenance, such as *Parkin* and *PINK1,* cause familial Parkinson’s disease (PD) [7]. *COA7* encodes a mitochondrial function-related protein, and its variants affect the stability and localization of proteins that interact with COA7, reducing the activity of mitochondrial complexes I and IV [1, 3]. *COA7* ribonucleic acid expression in the midbrain is not significantly higher than that in other brain regions (Human Protein Atlas, http://www.proteinatlas.org). However, the precise functional role of *COA7* is not fully understood [1, 2]. *COA7* gene abnormality might cause mitochondrial dysfunction, resulting in damage to the fragile dopaminergic neurons in the substantia nigra and the development of parkinsonism. Further, the DAT scan, which reflects dopamine neuron number and function in PD [8], showed decreased uptake in the bilateral striata, indicating dopamine neuron dysfunction or loss. The patient’s condition most likely developed during adulthood and progressed slowly, causing vulnerable mitochondrial function in dopaminergic neurons, thus triggering neuronal death and parkinsonism.

In our case, MIBG scintigraphy did not show decreased uptake; however, it reflected the status of the cardiac sympathetic nervous system. Early PD stages (H&Y stages 1 and 2) indicate a cardiac sympathetic denervated pattern, with a sensitivity of 94.1% and specificity of 80.2%, indicating a decreased heart-to-mediastinum (H/M) ratio on MIBG scintigraphy [9]. MIBG scintigraphy results revealed familial PD caused by mitochondrial maintenance-related proteins, showing that *Parkin* mutations are normal [10]. However, the *PINK1* heterozygous variant decreased the H/M ratio in 12 out of 23 cases (50%) [11]. Decreased uptake was not observed on scintigraphy in our case; however, the disease might be at an early stage before sympathetic nerve denervation occurred, or the sympathetic nervous system is preserved in *COA7*-variant cases.

Among the six reported cases [1–3], only one had the same homozygote variant (patient 1; c.17A > G), whereas two had compound heterozygous variants (patient 3; c.17A > G/c.446G > T and patient 4; c.17A > G/c.430delG) in *COA7*. In these cases, the age at onset was 5 years old or younger in two patients and 15 years in one patient, with no adult onset noted as in our case. Further, all three patients experienced sensory disturbance due to polyneuropathy, decreased tendon reflexes, and positive Romberg sign. The three patients did not have limbs and truncal ataxia, as observed in our case. However, all showed cerebellar atrophy on brain MRI, and parkinsonism was not reported in any case. A limitation of our study is that the onset of coincidental parkinsonism with idiopathic PD could not be ruled out.

In conclusion, this is a case of SCA with axonal neuropathy type 3 with *COA7* gene variants showing parkinsonism in an adult. There have been no reports on adult-onset SCA or parkinsonism with *COA7* gene variants; however, the functional imaging findings of reduced bilateral striatal uptake on DAT scan and normal MIBG scintigraphy are consistent with hereditary parkinsonism with mitochondrial dysfunctional gene mutation. Consequently, parkinsonian symptoms might be a symptom of diseases caused by *COA7* gene mutations. However, further studies must clarify whether parkinsonism is a symptom of *COA7* gene variants.

## Data Availability

Not applicable. The data used during the current study are available from the corresponding author upon reasonable request.

## Abbreviations

COA7: Cytochrome c oxidase assembly factor 7
CMT: Charcot-Marie-Tooth disease
SCA: Spinocerebellar ataxia
MRI: Magnetic resonance imaging
CSF: Cerebrospinal fluid
DAT: Dopamine transporter
MIBG: ^123^I-metaiodobenzylguanidine
PD: Parkinson’s disease
H/M ratio: Heart-to-mediastinum ratio
HTLV-1: Human T-cell Leukemia Virus Type 1
MDS-UPDRS: Movement Disorder Society-sponsored revision of the Unified Parkinson's Disease Rating Scale
